# The regulation of brain states by neuroactive substances distributed via the cerebrospinal fluid; a review

**DOI:** 10.1186/1743-8454-7-1

**Published:** 2010-01-06

**Authors:** Jan G Veening, Henk P Barendregt

**Affiliations:** 1 Dept of Anatomy, (109) UMC St Radboud, PO Box 9101, 6500 HB, Nijmegen, the Netherlands; 2 Dept of Psychopharmacology, UIPS, Univ of Utrecht, the Netherlands; 3 Fac of Science, Radboud University, Nijmegen, the Netherlands

## Abstract

The cerebrospinal fluid (CSF) system provides nutrients to and removes waste products from the brain. Recent findings suggest, however, that in addition, the CSF contains message molecules in the form of actively released neuroactive substances. The concentrations of these vary between locations, suggesting they are important for the changes in brain activity that underlie different brain states, and induce different sensory input and behavioral output relationships.

The cranial CSF displays a rapid caudally-directed ventricular flow followed by a slower rostrally-directed subarachnoid flow (mainly towards the cribriform plate and from there into the nasal lymphatics). Thus, many brain areas are exposed to and can be influenced by substances contained in the CSF. In this review we discuss the production and flow of the CSF, including the mechanisms involved in the regulation of its composition. In addition, the available evidence for the release of neuropeptides and other neuroactive substances into the CSF is reviewed, with particular attention to the selective effects of these on distant downstream receptive brain areas. As a conclusion we suggest that (1) the flowing CSF is involved in more than just nutrient and waste control, but is also used as a broadcasting system consisting of coordinated messages to a variety of nearby and distant brain areas; (2) this special form of volume transmission underlies changes in behavioral states.

## Review outline

Introduction

Production and circulation of CSF

Production and sources of CSF

Flow and destiny of the CSF

The CSF circulation during development and aging of the CNS

CSF composition: sources, targets and exchange with ECF

Sources of CSF contents

Flow-transport: speed and mechanisms

Exchange of substances between CSF and ECF; mechanisms and targets:

Neuropeptides form an integral part of the CSF contents

Behavioral effects of CSF contents

Conclusions

## Introduction

Behavioral observations as well as modern imaging techniques show that brain activity is an input-output process that depends on the physiological or behavioral state of the animal or subject. A state is a well-known concept in mathematical system theory and in computer science. A machine, but for that matter also an organism or even a human, is at moment t_1 _in the same state as at moment t_2_, if for all possible input, the resulting internal and external reactions are the same. The states will be different if there is a particular input resulting in different reactions. Of course, the collection of all possible input (and also reactions) is huge and in a particular scientific discipline one restricts oneself to a set of relevant inputs and reactions. Many well-known examples of states come from animal observations, and have been described as motivational, emotional and mental states, or simply brain states in animal and human behavioral studies [1-5] and in a phenomenological study [6].

Changes in behavioral states, which induce adaptive physiological mechanisms, require a complex rearrangement of neural activity in a wide variety of brain areas. The hypothalamus, embedded in the limbic system is strongly involved when a motivational state changes [3-5,7,8]. Other brain areas, such as cortical regions or spinal cord, must be involved as well, in order to accomplish coordinated adaptations in the neural (sensory, motor and autonomic) as well as hormonal systems, that underlie goal-directed behavior.

Neural networks involved in the coordination of these changes are complex. Synaptic communication plays a role whenever rapid changes in neural activity are necessary. However, non-synaptic or paracrine communication has been proposed as a mechanism to coordinate changes in neuronal activity in multiple brain regions, for a prolonged period of time [5,7-11]. These mechanisms, synaptic and non-synaptic, may serve the same goal, however, and are usually well coordinated, complementary and mutually supportive, as will be discussed later. All changes induced by these mechanisms can be viewed as basic requirements for motivational changes. Many neuropeptidergic fiber systems descend over long distances, are abundantly present in behavior-relevant brain areas, contain mostly unmyelinated varicose fibers and lack synaptic contacts [7-15]. This combination of characteristics puts these fibers in a most favourable position to participate in changing the weight factors in the neural network involved in maintaining or switching between behavioral states [4,5]. In 1985, Nieuwenhuys [9] wrote (p 197): "I concur with the opinion of Chan-Palay that the periventricular part of the neural extracellular space presumably communicates via ependymal elements with the ventricular system, which in its turn may also represent an important communication channel, acting as a vehicle for widespread distribution of neuroactive substances". From 1986, the term volume transmission has been used for this type of non-synaptic communication [16-21].

Many descending fibers showing paracrine characteristics are observed close to the ventricular system [12,14,15,22], which raises the question as to how far the cerebrospinal fluid (CSF), flowing through the ventricular system and the subarachnoid space surrounding the brain, could be involved in behavioral changes. As early as 1910, Cushing and Goetsch [23] discussed the CSF circulation as a possible way to send messages to distant parts of the brain. Using CSF, obtained from patients with a brain tumour causing obstructive hydrops, strong physiological reactions were observed in rabbits after CSF injections. Later on, Cushing coined the term "third circulation" for the CSF flowing caudally through, as well as around, the CNS. In 1932 Friedman and Friedman investigated the effects of CSF oxytocin on rabbit uterus contractions and noted that the concentrations were effective [24]. Many years later, Borison *et al*. noted that "the simple concept that the subarachnoid space constitutes a catch basin for interstitial fluid is no longer tenable. Rather, the CSF of that space must be considered as being in dynamic chemical communication with all parts of the central nervous system" [25]. In 1998 a special meeting was held in Los Angeles about a possible role of CSF in spreading the messages and Nicholson reported on signals that go with the flow [26]. In the present review we explore the hypothesis that neuropeptides can be released into the CSF as messages to flow with the fluid, in order to induce changes in motivational states, by influencing appropriate receptors in distant brain areas.

The reviewed data concerning the CSF flow and the distribution of messages support earlier proposals by Sewards and Sewards [27,28] concerning vasopressin and corticotrophin releasing factor (CRF). In a recent paper, the presence of neuroendocrine signalling molecules in the CSF, led to the conclusion that they play an active role in the function of the nervous system [29], in full agreement with our proposal.

### Production and circulation of CSF

**Figure 1:**
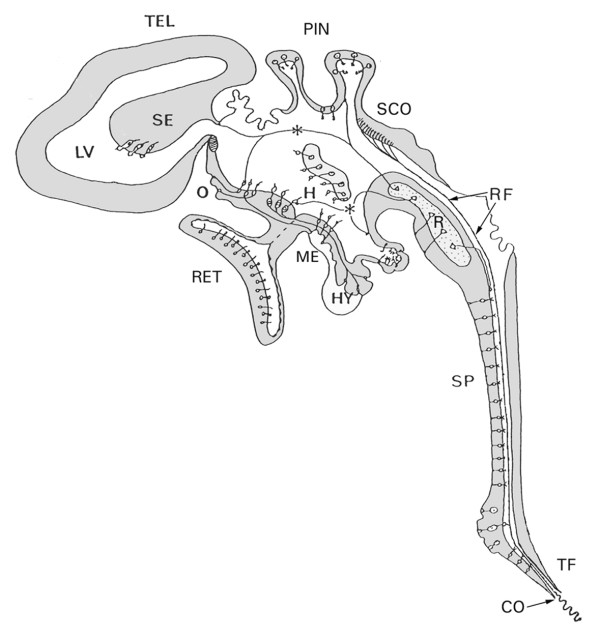
**A schematic diagram of structures and specialized cell types bordering the different parts of the mammalian ventricular system, and in contact with the cerebrospinal fluid (CSF)**. The complexity of the system suggests that CSF functions are not limited to metabolic support of the brain and the release of waste products. Abbreviations: **CO**: caudal opening of the central canal of the spinal cord;**H**: hypothalamic CSF-contacting neurons; **HY**: Hypophysis; **LV**: lateral ventricle; **ME**: median eminence; **O**: vascular organ of the terminal lamina; **PIN**: pineal organ; **R**: raphe nuclei; **RET**: retina; **RF**: Reissner's fiber; **SE**: septal region; **SCO**: subcommissural organ; **SP**: medullo-spinal CSF-contacting neurons; **TEL**: telencephalon; **TF**: terminal filum; (Fig. 1 was kindly provided by Prof. B. Vigh. For details about specific cell types, the reader is referred to: Vigh and Vigh-Teichmann, [31], and to Vigh *et al*, [32]).

Anatomically, the ventricular system of the vertebrate brain develops from a simple elongated cavity inside the neural tube, into a highly complex structure, filled with CSF (Fig 1) [10,30]. Vigh and others have extensively studied the specialisations and CSF-contacting neurons composing and surrounding the ventricular walls [31-35].

The CSF has an essentially unidirectional flow [36]; caudal through the ventricular system and in different directions through the cisternal subarachnoid spaces surrounding the brain, providing the brain with a protective environment. The CSF can be characterized by its open communication with the extracellular fluid (ECF) of the neuropil, to such an extent that CSF has been described as a reservoir of cerebral ECF [37]. The intercellular junctions between the ependymal cells determine the extent of this communication, and there are many regional differences [32]. In addition, recent investigations suggest that this open communication is biased in the sense that CSF has rapid and widespread access to the ECF, causing local interstitial oedema when CSF-pressure is rising, like after acute hydrocephalus [38,39]. In the opposite direction, however, only about 10 to 15% of the ECF drains into the CSF [40,41]. The intercellular junctions in the superficial and perivascular glial limiting membranes may play a role here by providing a separation between subarachnoid and perivascular CSF space and brain ECF.

Therefore, CSF-ECF exchange of neuroactive substances is a variable and complicated process. Concentrations vary over time and in addition there may be large regional concentration differences of substances. Such differences arise following massive local release of substances in the brain, but exist in the CSF as well, where concentrations can vary by a factor of 10 in different regions of the ventricular system [36,42].

CSF has a high salt concentration (>150 mmol/l) and low protein concentration (ca. 200-700 μg protein/ml). It serves as a transport medium, carrying nutrients for cells and removing products of their metabolism. However, CSF not only contains polypeptides which pass through the blood-brain barrier but also harbours peptides and proteins manufactured locally. The CSF protein population differs in composition from that of plasma due to inherent CSF functions, such as ongoing proteolytic processes involved in cell surface remodeling, protein shedding, and synthesis of regulatory peptides [36,43,44]. Blood-derived labelled proteins, administered peripherally, appear first in the lumbar CSF and only later in the cisternal or ventricular CSF [36], while their concentration remains highest in the lumbar subarachnoid space. The additional 20% of the CSF peptides are brain-derived, however, and have the highest concentration in ventricular CSF steadily decreasing along the flow pathway through the subarachnoid space [36]. Apparently, gradients in CSF levels exist and concentration differences of such peptides along the rostrocaudal axis of the central nervous system tell us something about their sources. In summary, the CSF shows an essentially unidirectional flow and may contain brain-derived peptides moving with the flow.

#### Production and sources of CSF

In the human brain, about 75% of CSF is secreted by the choroid plexuses inside the lateral ventricles [45]. The additional 25% is contributed by the ECF across the ependymal layer [46]. CSF production in the young human brain is about 500 ml/day or about 340 μl/min [40,45]. This reduces to about 250 ml/day for the elderly, [47]. The adult brain contains a constant volume of about 140 ml of CSF, of which 30 ml occurs in the ventricles and about 110 ml in the subarachnoid spaces [40]. Therefore, human CSF is completely replaced about three times every 24 hours. In the young adult rat, which has about 200 μl CSF, it takes about 2 hours to replace the CSF whereas in 30 month-old rats with 300 μl, it may take up to 8 hours for complete replacement [48].

The production of CSF is under control of factors working on both sides of the choroid plexus [29]. On the vascular or basal side of the epithelium, noradrenergic and cholinergic nerves may exert direct control, diminishing or increasing production, respectively, as reviewed by Perez-Figares *et al *[46]. On the ventricular side substances present in the CSF may influence receptors on the apical surface of the choroid plexus epithelium. Serotonin, for example, is probably released into the CSF by the supraependymal plexus (see below) and regulates CSF production via binding to 5-HT_2C _receptors, thereby reducing CSF secretion. Similar mechanisms have been proposed to explain the effects of dopamine, norepinephrine, melatonin and several peptides, among them vasopressin (See [46] for review).

In addition, the composition of CSF is dependent on the activity of surrounding brain tissue [29,36] and on the activity of a specific circumventricular (CVO) organ, the subcommissural organ and Reissner's fiber complex (Fig 1) [49,50]. Reissner's fiber develops and extends from the subcommissural organ in a caudal direction, eventually reaching and even extending from the caudal tip of the central canal of the spinal cord (Fig 1) [51,52]. In the adult human brain, the subcommissural organ and Reissner's fiber can no longer be detected [52], suggesting that the fiber plays a special role during the development of the mammalian CNS [50]. This fiber is composed of aggregated glycoproteins and has the capacity to bind substances present in the CSF and most probably affects the clearance of monoamines from the CSF [53,54].

The subcommissural organ itself is under serotonergic control [55], while the choroid plexus is under control of the autonomic nervous system, which in turn is controlled by a number of hypothalamic regions. These circumstances make it clear that CSF production and composition is under the influence of an extensive set of regulatory brain mechanisms. In addition, changes in CSF- and intracranial pressure or a developing hydrocephalus seem to be involved in regulatory feedback control mechanisms [56]. In summary, both the production and the composition of the CSF are controlled by a variety of mechanisms.

#### Flow and destiny of the CSF

CSF is constantly flowing inside the central nervous system, driven by the arterio-venous pressure gradient and the secretory processes at the choroid plexuses [36]. Two different kinds of flow, bulk flow and laminar flow, have to be distinguished, since the mechanisms are very different and probably subservient to very different functions.

The bulk of the CSF flows in a caudal direction via the third ventricle and the cerebral aqueduct to the fourth ventricle. From there it flows either via the lateral apertures of Luschka in all sub-primate vertebrates, and via the additional median aperture of Magendie in primates only [57] into the subarachnoid space including cisternae magna and pontis, surrounding the brainstem [58,59]. Some flow may continue through the central canal of the spinal cord but most of the subarachnoid CSF flow apparently divides in the cisterna magna into a cortical, rostrally directed, and a lumbar, caudally directed, component [36]. Inside the ventricular system, turbulences can occur at the entrance to the narrowest parts such as the cerebral aqueduct and central canal, which may hinder the flow of the CSF. Such turbulences are probably reduced by the presence of Reissner's fiber [60]. In the central canal of the rat spinal cord, CSF has been observed to continue flowing caudally at a speed of about 1 cm/min [60]. At the caudal tip of the spinal cord in the filum terminale, one opening (rat) or several openings (guinea pig, rabbit) (Fig 1) allow the CSF to access the subarachnoid space [61]. The CSF surrounding the spinal cord may either rapidly re-enter the spinal cord and central canal via perivascular spaces [62], or leave perispinal spaces to be released into lymphatic or venous vessels [63]. Recent human studies suggest that spinal CSF absorption via perispinal spaces alongside the exiting spinal nerves may account for one third of total CSF absorbed in the resting state and considerably more in active individuals [45].

**Figure 2:**
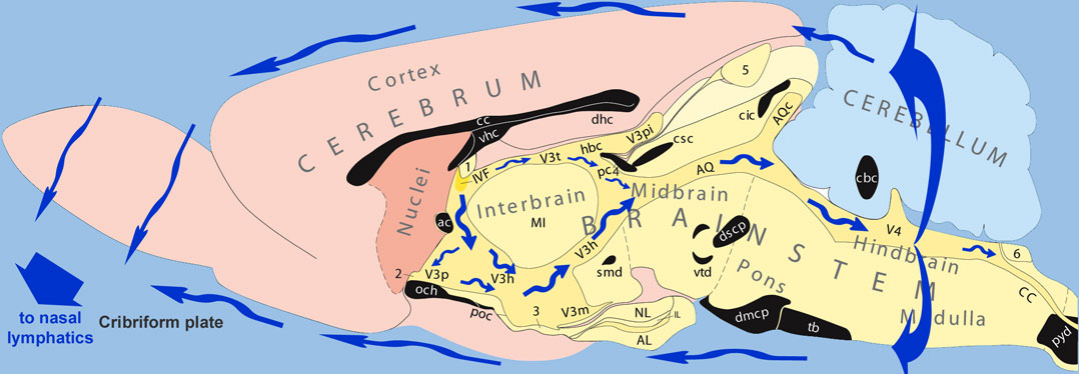
**A schematic diagram showing the direction of the CSF bulk flow in the mammalian brain based on a midsagittal section of the rat brain, kindly provided by Prof. L.W. Swanson**. The following regions are indicated: telencephalon (pink shades); diencephalon (interbrain) and brainstem areas (yellow shades); cerebellum dorsal to the brainstem (light blue); sectioned fiber tracts (black). CSF (blue arrows) flows from the lateral ventricles to the third ventricle via the interventricular foramen (IVF), and flows caudally along the dorsal and ventral side of thalamic adhesion, to the cerebral aqueduct (AQ) and fourth ventricle (V4). Some CSF may continue flowing caudally through the central canal of the spinal cord (CC), but most leaves the ventricular system via the lateral apertures and flows through the subarachnoid space, surrounding the brain. This external flow is indicated here along the dorsal and ventral side of the brain but occurs also along all other external brain surfaces. The destination of the subarachnoid flow is the cribriform plate of the ethmoidal bone, containing the penetrating olfactory fibers, where CSF is released in small lymphatic vessels. Additional abbreviations: V3(p, h, m, t, pi): regions and recesses of the third ventricle; Circumventricular organs: 1: subfornical organ; 2: organum vasculosum of the lamina terminalis; 3: median eminence; 4: subcommissural organ; 5: pineal organ; 6: area postrema; AL, IL and NL: different lobes of pituitary; Fiber bundles crossing the midline, coloured black, are not relevant for the present review.

Concerning the cranial CSF surrounding the brain, most textbooks tell us that arachnoid granulations, penetrating the dura and extending into the superior sagittal sinus, compose the main outlet system for CSF absorption in the human. Recent animal investigations, however, have shown that at least 50% and perhaps up to 80% or more of the CSF leaves the cranial cavity through the cribriform plate, via the perineuronal spaces surrounding the olfactory nerves, to drain into the nasal and cervical lymphatics (Fig 2) [64-70]. This rostral outflow of CSF contains considerable amounts of interstitial fluid, especially from midbrain areas [71,72], and plays an important role in CNS immune system interactions [68,73].

The flow of CSF is indicated in Fig 2. Additional CSF absorption pathways may exist alongside the vessels of the cavernous sinus and via small lymphatic vessels emerging from the perineuronal space of other cranial nerves, like the trigeminal nerve [69]. These cranial CSF exits operate in parallel to the spinal CSF exits to the lymphatic system, mentioned before [63]. Bringing these data together, it seems to be reasonable to hypothesize that of all CSF produced continuously in the human brain, about one third leaves via the spinal vessels and one third via the nasal lymphatics, which leaves about one third for absorption through the arachnoid granulations into the superior sagittal sinus. The balance between these absorption pathways may vary considerably, however, depending on body position, physical activity and other possible factors.

The driving force of the CSF bulk flow is actively supported by arterial pulsations as proposed by Bering [74], in the choroid plexus or by arterial and brain expansion mechanisms [75-78]. A decade ago, these temporarily neglected expansion mechanisms started to receive attention again because of potential clinical relevance [46,79-83] and age related changes [84].

The laminar flow of CSF occurs in a thin layer along the ventricular walls and is not necessarily restricted to directions from rostral to caudal. Directional beating of ependymal cell cilia lining the ventricular wall, seems to be the most important factor for this flow [60,85,86]. The ciliary movements may contribute to the mixing of the CSF, are supported by a sialic acid-induced hydration mantle on the ventricular surfaces and are stimulated by release of serotonin from the supraependymal serotonergic terminals. In addition to transport of neuroactive substances, a rostrally-directed laminar flow may be crucial for newborn neurons to reach their final location (see below). Both bulk and laminar flow seem to be prerequisites for normal CSF functioning, since disturbance of either the ciliary movements or Reissner's fiber both perturb CSF flow to such an extent that hydrocephalus develops [87,88].

In summary, the flow of the CSF comprises two different mechanisms. Bulk flow is driven by arterio-venous pressure gradients and arterial pulsations, traversing the ventricular system in a caudal direction towards the brainstem apertures. From there the subarachnoid CSF flow can be described as bidirectional: either descending to the spinal cord or ascending to the dorsal and rostral parts of the brain. Since in the latter, cranial, compartment the main exit of the CSF appears to be along the olfactory nerves through the cribriform plate, there must be an important rostrally directed vector in this subarachnoid CSF flow. The laminar flow occurs only inside the ventricular system in a very thin layer along the walls in a variety of directions and driven by the beating of the cilia of local ependymal cells. Interruption of either bulk or laminar flow results in hydrocephalus.

#### The CSF circulation during development and aging of the CNS

Recently, it was shown in mice that ependymal ciliary beating, inducing laminar flow, is a prerequisite for the migration of new neurons from the subventricular zone to the anteriorly located olfactory bulb [89]. Even in the adult human brain, newly formed neuroblasts follow this path between the subventricular zone and the olfactory bulb. This path is referred to as the rostral migratory stream [90]. Apparently, supported by gradients of brain-derived neurotrophic factor (BDNF) levels in the CSF [91], the neuroblasts know how to go with the laminar flow.

Experimental evidence suggests that during development of the brain, messages from specific parts of the ventricular system are essential for the formation of the layered structure of the cortex [92]. This suggests that specific messages released into the CSF at a specific time point, influence not only functional but also important structural aspects of distant target areas. In addition, such findings suggest that the targets for CSF messages may not be limited to the periventricular areas inside the brain but may be present at the outer surface of the brain as well.

The features of the walls lining the ventricular system also change over time. In the rat brain, ependymal cells appear from gestational day 16 [93]. The function of the ependymocytes as well as the permeability of the ependyma is age-dependent [94-96] and contributes to the changes with age in composition of the CSF [48,96]. In elderly humans CSF production and flow rate decrease, affecting protein concentrations [36] demonstrating that clear functional changes occur in the third circulation with increasing age.

Clinical evidence shows that changes occurring in the composition of the CSF and in the homeostatic balance of freely exchanging CSF and ECF compartments with age [36], are possibly involved in brain diseases such as Alzheimer's [48,97]. On the one hand, such changes in the composition of the CSF may profoundly affect the paracrine messages transmitted via the CSF. On the other hand, it may facilitate new opportunities for the treatment of brain diseases as prospects are improved for drug delivery and brain targeting via the CSF for multiple brain diseases [98]. In summary, CSF flow plays an important role in the structural development of the CNS. The laminar flow is involved in the guidance of neuroblasts and the bulk flow in delivering messages obtained from the brainstem to guide cortical development. The composition of CSF and CSF-ECF exchange are profoundly affected by the aging process and may be related to brain diseases, suggesting an opportunity for treatment by targeting drugs via the CSF.

### CSF composition, sources, targets and exchange with ECF

#### Sources of CSF contents

In addition to blood-derived proteins in the CSF (approximately 80%), the other proteins (about 20%) originate from within the CNS, from neurons, glial and leptomeningeal cells [36]. The choroid plexus itself synthesizes proteins, some of them abundantly, such as transthyretin, a carrier for thyroid hormones. Once secreted into the CSF, such proteins are thought to influence distant parts of the brain [99]. The direct or indirect activation of brain areas bordering the ventricular system or subarachnoid space has been shown to result in increased levels of neuropeptides released into the CSF [100-102]. The circadian variations in CSF concentrations without concomitant changes in peripheral concentrations provide additional evidence for selective release of neuropeptides such as oxytocin into the CSF [103-106]. Specific dendritic release mechanisms occur in brain areas such as the paraventricular and supraoptic hypothalamic nuclei, leading to as much as a thousand fold increase in the local ECF peptide concentrations [107]. Experiments involving ablation and transplantation of the suprachiasmatic nucleus in the golden hamster showed that the release of a diffusible signal was most likely carried by the flow of CSF [108,109]. Recently, it has been shown that in the diurnal grass rat, the suprachiasmatic nucleus releases at least some of its contents into the CSF in order to reach specific target areas [110]. Taking together the available evidence, we suggest that the active release of neuropeptides into the CSF has to be considered as a probable mechanism for message transmission via the CSF to distant brain areas, as was previously discussed during a special conference in 1998 [26].

**Figure 3:**
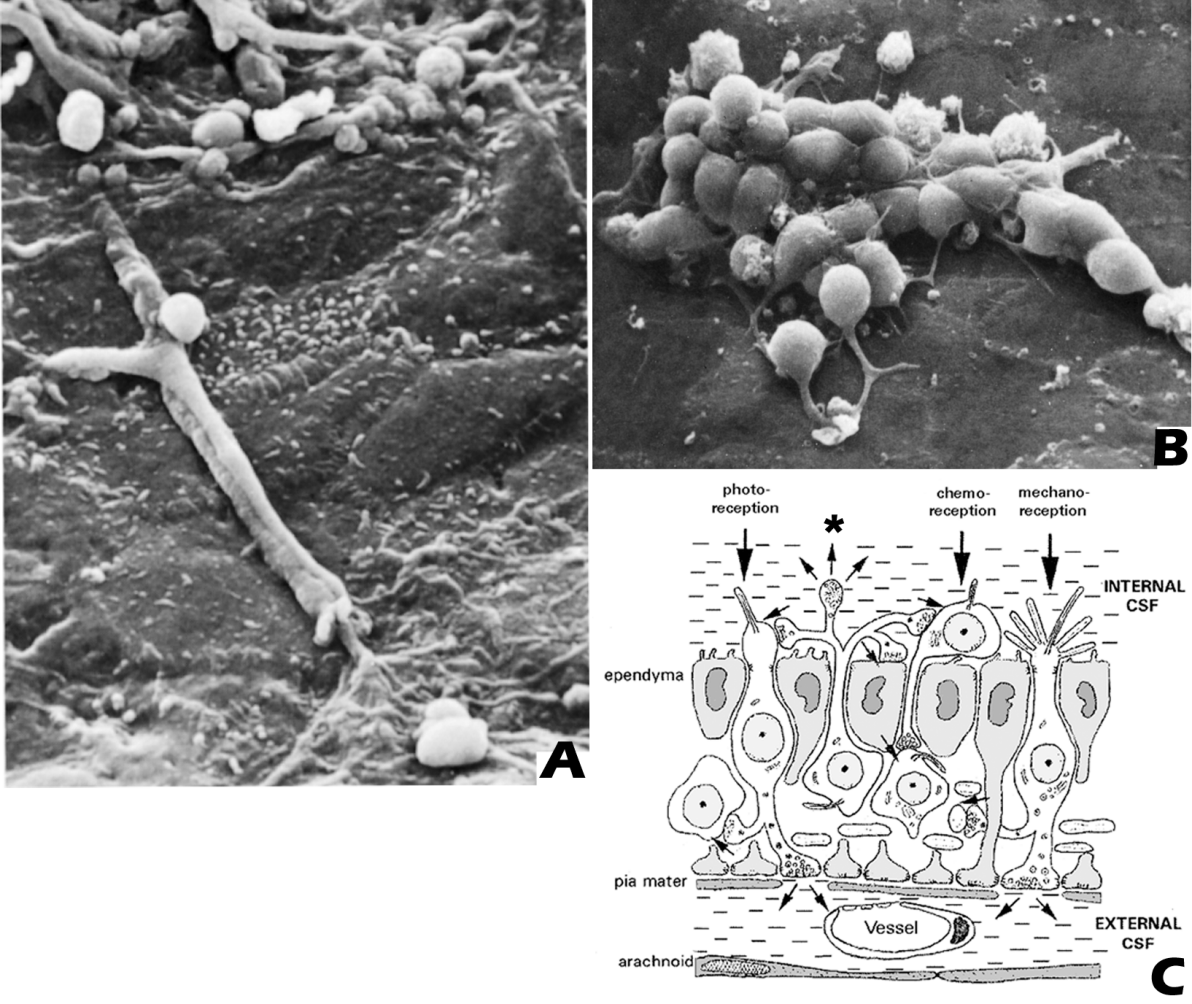
**(A and B) show scanning electron microscopy of supraependymal structures in the rat brain**. A: Partially ensheathed fibers and terminal arborizations; B: cellular structures with short fiber extensions, on the ventricular surface of the ependymal cells; Fig 3C: a schematic diagram of the ependymal layer with supra- and subependymal cellular structures contacting the CSF at the ventricular side of the layer; the * indicates a supraependymal terminal extending into the CSF, as described for serotonergic fibers. Figs 3A and 3B were reprinted from [121], with permission of Springer-Verlag. Fig 3C was kindly provided by Prof. B. Vigh.

In addition to the neuronal release from specific brain areas, the presence of widespread supraependymal cell clusters, fibers and plexuses of neuronal and other cellular elements, including macrophages, have been described since the early seventies [32,111]. Their abundant presence near the median eminence suggests that these supraependymal elements are involved in a variety of functions [112-117] (Fig 3A, B). Among these, serotonergic fibers have been described as originating from the raphe nuclei with nerve terminals located in the CSF. These fibers are thought to release their content differentially from several parts of the ventricular walls of the mammalian ventricular system into the flowing CSF [111,112,117-121] (Fig 3C).

In addition to the supraependymal elements, many fibers containing specific neuroactive substances can be observed in the subependymal layer. Many of these are of hypothalamic origin and may contain peptides like luteinising hormone releasing hormone (LHRH), CRF or adreno-corticotropic hormone (ACTH) [12,14,122]. Others originate in the dorsal periaqueductal gray to descend towards the cervical and upper thoracic spinal cord, many of them contacting the perivascular spaces [22]. Many of these descending fibers are characterized by large numbers of varicosities or local swellings containing numerous vesicles but without synaptic specialisations. The presence of such varicosities suggests that a single fiber may release its contents over large distances, by non-synaptic release or exocytosis, either into the CSF or for diffusion into the bordering ECF to influence a population of receptive neurons [12,14,31,32].

From the available data, no clear general function can be derived for these supra- and sub-ependymal elements. However, their differential distribution over specific regions of the ventricular walls strongly suggests two possibilities: local effects and/or specific effects on receptive distant target areas. Two interesting cases of substances released into the third ventricle, going with the flow to reach their target areas, have been studied in detail so far: melatonin and the LHRH system, also known as the gonadotropin-releasing hormone (GnRH) system.

The pineal gland produces the hormone melatonin at night. The gland is located dorsal to the third ventricle and has access to the CSF via the pineal recess [123]. In a series of experiments in sheep, it was shown that the main effects of the pineal gland occurred through release of melatonin into the dorsal tip of the third ventricle. At the exit of the pineal gland, concentrations were about 10 times higher than in the rest of the ventricular system and about 100 times higher than in peripheral blood [123,124]. Melatonin is not only secreted into the CSF but also into the general circulation [124,125], which makes the situation more complex. Melatonin also regulates the CSF levels of another hormone, estradiol [126], which may result in additional indirect effects. However, its main site of action to control reproduction is located in the premammillary hypothalamic nucleus, where it stimulates luteinizing hormone secretion in the ewe [127]. Apparently, transport mechanisms from the pineal gland via the CSF to the premammillary hypothalamus are involved.

Another example is posed by the neuropeptide: LHRH also known as GnRH. This neuropeptide is released in a pulsatile manner into the portal system and stimulates the pulsatile gonadal hormone production of the pituitary [128,129]. However, GnRH is released into the CSF as well, and this CSF GnRH neither functions as a feedback system for controlling GnRH production, nor does it directly affect the pituitary gland [130]. The influx of peripheral GnRH across the blood-brain barrier into the CSF is so limited that it cannot explain the observed levels of CSF GnRH which are comparable to those in the portal blood stream [131]. These findings, combined with detailed measurement of GnRH concentrations at different sites in the ventricular system suggest that GnRH is released directly into the CSF and the third ventricle. This occurs at the level of the median eminence, and possibly the organum vasculosum of the lamina terminalis (OVLT) (indicated as ME and O in Fig 1) where GnRH immunoreactive (IR) perikarya and axons are very abundant [128,131]. The authors conclude that the function of CSF GnRH expresses itself in behavioral effects, induced by volume transmission via the flow of CSF to affect distant brain areas, such as periaqueductal gray (PAG) and hippocampal regions. These regions contain, indeed, high densities of GnRH receptor expressing neurons, while GnRH-IR axons are either scarce (PAG) or completely lacking [12,15,131]. The PAG is strongly involved in sexual behavior [132-135] which can be elicited by intraventricular (icv) but not by intravenous administration of GnRH [136]. These findings concerning the distribution and behavioral effects of melatonin and GnRH via the CSF comprise, in our view, compelling evidence for CSF messages controlling aspects of behavior.

That the exact site of release of a neuropeptide into the ventricular system is the determining factor for its given effects, has been shown in experiments involving transplants of the suprachiasmatic nucleus in the hypothalamus [109,137]. Research aimed at identifying the mechanisms regulating food intake, such as experiments involving delivery of substances into either the third or the fourth ventricle, with an open or an experimentally-closed cerebral aqueduct, have shown that caudally-flowing substances and neuropeptides (glucose, leptin, neuropeptide tyrosine (NPY) reach specific receptive areas to influence food intake [138-140]. Apparently, most factors, influencing food intake, like cocaine- and amphetamine-regulated transcript (CART), bombesin and oxytocin, exert their effects in the caudal brainstem [141-144], while other factors are only effective in or around the third ventricle (NPY and calcitonin [140,145].

In summary, about 80% of the CSF proteins are derived from the blood [146] while about 20% are produced by the brain. Brain areas bordering the ventricular system as well as supra- and subependymal cells and fibers lining the ventricular walls are in the most favourable position to add such substances to the CSF. Melatonin and GnRH, both released into the third ventricle but at different locations, and the experiments concerning substances controlling food intake administered into either the third or the fourth ventricle, and moving with the flow, are good examples for local additions into the CSF influencing remote brain areas. Apparently, the site of upstream release determines which downstream areas will be affected.

#### Flow-transport: speed and mechanisms

The question can be raised whether the flow-transport mechanisms work rapidly enough to induce behavioral effects in a relevant timescale. A quick survey of the literature shows that the time between administration or release of neuropeptides into the CSF and the observed behavioral or physiological effects turns out to be very short. In a series of experiments involving respiratory and cardiovascular control mechanisms in the cat, effects of icv administration of respiratory stimulants such as vasopressin into the lateral ventricle were observed within two minutes at the ventral surface of the brainstem. Since this surface lies beyond the foramina of Luschka, this finding indicates that the stimulus was moving with the CSF flow in the cat brain at a speed of more than 1 cm/min [57]. A tracer like HRP carefully administered into the lateral ventricle of the cat in order to avoid direct pressure effects, penetrates the outer surface of the brainstem within four or five minutes (see below) [25]. A similar rapid distribution of a tracer [^14^C]Inulin as well as of [^125^I]CRH after administration into the lateral ventricle has been observed in the rat [147]. Other data obtained from the rat also show rapid flow transport of neuropeptides. α-Melanocyte stimulating hormone (αMSH) injected into the lateral ventricle appeared within two minutes in the cisterna magna [148]. β-endorphin was observed in the CSF within minutes after electrical microstimulation of the arcuate hypothalamic nucleus, without any change in peripheral levels [100,149]. Both interleukin-1 and CRF have been shown to induce widespread effects throughout the brain, starting a few minutes after icv administration [150,151]. The effects of icv oxytocin on ventromedial hypothalamic neurons is manifest in under one minute [152]. Fenstermacher and his group showed a similar rapid distribution via the ventricular system, however their findings revealed that the flow over the cortical surface was considerably slower [146,153,154].

Recent measurements of the pulsatile flow rates in the cerebral aqueduct of the human brain by modern imaging and other techniques fully support the high speed of this CSF transport channel [75,78,83]. Maximal flow rates of 12 mm/s and higher were observed in the aqueduct [58,59], while at other sites like the spinal canal, the flow rate was almost negligible [58].

In summary, transport of CSF contents by bulk flow is fast, working on a time scale of minutes, and is directly relevant for behavioral and physiological adaptations.

#### Exchange of substances between CSF and ECF: mechanisms and targets

Exchange of fluid contents between the ECF in the neuropile and the ventricular CSF occurs via different mechanisms and at different rates: 1: diffusion, 2: perivascular pump mechanisms, and 3: tanycytes and ECF-contacting neurons.

1: Diffusion. This is a rather slow process, depending on ECF diffusion parameters like volume fraction, tortuosity and the diffusion coefficient of the neuroactive substance [155-157]. Diffusion in the ECF compartment may appear anisotropic, due to the hindrances imposed by local differences in tortuosity [155]. White matter tracts are guiding tracts for the interstitial fluid flow as well as for the dissemination of primary brain tumors [158]. Diffusion and exchange between cerebrospinal and extracellular fluid compartments may occur in both directions [37,77] through an uninterrupted single-layered lining of the ventricular system, consisting of several types of ciliated ependymal cells [95,159]. Due to the lack of tight junctions between ependymal cells, the CSF-ECF barrier can be described as open or non-existent. In that respect, the CVO's form a notable exception with a closed CSF-ECF barrier and an open blood-ECF barrier (usually called blood-brain barrier, or BBB), (see McKinley et al[160]). Interestingly from a cybernetic point of view, the open CSF-ECF compartment throughout the brain is functionally separated from the open BBB-ECF compartment inside the CVO's. These two compartments can be described as being disjoint or even complementary, but the direct neuroanatomical relationships between the paraventricular hypothalamic nucleus and the CVO's [161-168] manage the bidirectional communication and mutual tuning of both compartments.

In general, concentration differences and gradients determine the direction of the diffusion between CSF and ECF. High levels of neuroactive substances in the CSF will enter the ECF, while high local concentrations in the ECF will diffuse into the CSF. For brain areas like the medial hypothalamus, as well as the periaqueductal gray, such diffusion processes will result in continuous equilibration processes in the levels of neuroactive substances involved in the induction or maintenance of behavioral states. Widely divergent substances and techniques have been used to measure diffusion distances. Diffusion over distances of 0.5 mm and at other sites up to 1 mm away from the wall of the third ventricle, from the cerebral aqueduct and from the floor of the fourth ventricle [25,147,151,169,170] are reasonable estimations. In rat brain, areas such as the lateral septum and parts of the bed nucleus of the stria terminalis, the dorsal midline thalamus and habenular nuclei, the medial hypothalamic regions, the mesencephalic periaqueductal gray as well as several brainstem core and paracore areas [7] can, therefore, be influenced directly by the composition of the CSF. Diffusion of any relevant substance from the CSF into these regions would influence a number of behavior-relevant brain functions [27].

Diffusion in the other direction, that is from the ECF into the CSF occurs as well and may also play a role over larger distances. This situation was observed after infusion of a small amount of beta-endorphin into the striatum of the rat [171]. Despite the considerable distance to the ventricular system, the infused beta-endorphin appeared in the CSF within 10 minutes, reaching peak values in the cisterna magna after about 30 minutes [171]. This experiment shows that diffusion within the brain may occur over considerable distances and in a behaviour-relevant time-scale during the CSF-ECF exchange processes.

On the other hand, many different substances have been injected into the ventricular system of a variety of vertebrates and a speed and depth of penetration has been observed which far exceeds the capabilities of diffusion. Apparently, active transport processes are involved as well. Two of these will be described.

2: Perivascular pump mechanisms have been shown to play an important role in the distribution of CSF contents. The pump involves the perivascular space of vessels penetrating the outer surface of the brain and is driven by the arterial pulsations of the cerebral vessels [172,173]. The differences between the passive diffusion processes are striking. While the diffusion rate of the neuroanatomical tracer horseradish-peroxidase (HRP) in brain tissue amounts to less than 1 mm/hr, after an intraventricular or cisternal injection, HRP can be observed along all microvessels of the neuraxis. HRP molecules penetrated deeply (several mm) within five minutes [172]. This fast spread occurs along perivascular spaces and is aided by a paravascular fluid circulation system, driven by the pulsations of the cerebral arteries. In parallel to an inward flow along the arterial perivascular spaces, an outward flow may occur along the venous paravascular routes, adding interstitial fluid to the ECF/CSF in the subarachnoid space [172,174]. The existence of these paravascular routes provides the neuroactive CSF contents with a widespread and fast portal to enter less superficial brain areas and to affect a much larger variety of regions in brain or spinal cord [62,175,176]. A more recent study, in which the tracer [^14^C]inulin was used, confirmed the rapid and extensive distribution of icv-injected substances. Interestingly, it was concluded that the paravascular fluid circulation may even preferentially target specific brain areas like the hippocampus, the basal forebrain including the hypothalamus and the ventral brainstem surface [147]. This perivascular pump has been described as a "powerful mechanism for the distribution of therapeutic molecules within the brain" [173]. Similar mechanisms may support neuropeptides released into the CSF to reach remote destinations. GnRH, for example, could reach the hippocampal regions, which are richly supplied with GnRH receptors while a GnRH innervation is virtually lacking [131].

3: In addition to the slow diffusion mechanisms for local/regional effects and the rapid perivascular pump mechanisms, involving the whole or some preferential large parts of the brain, special ependymal cells (tanycytes) and neurons contact the CSF in order to internalize specific constituents, to release them at specific sites and for specific purposes, and are not covered by the general mechanisms mentioned before.

Tanycytes compose a specific group of ependymal cells. Those lining the ventral part of the third ventricle have basal processes that extend to the hypophyseal-portal vasculature as well as into the mediobasal hypothalamus, suggesting a role in pituitary and neural control. As reviewed by Bruni [95] and Rodriguez *et al*. [177], multiple tanycyte functions have been proposed over time. Tanycytes can be observed also in the walls of the lateral and fourth ventricles as well as along the central canal of the spinal cord [178]. Tanycyte extensions apparently radiate from the third ventricle into hypothalamic nuclei, but also laterally into the caudate putamen or, from the floor of the fourth ventricle, ventrally into the lower brainstem areas [178]. Several types of tanycytes have been described with specifically different endocytotic mechanisms [179,180]. Some of these recent experiments compared peripheral and icv administration of tracers and neuropeptides and the distribution and tanycyte-labelling patterns inside the brain were very different [179,181]. This suggests that tanycytes of a given type are specifically selective, each one retrieving a different substance from the CSF and underlining the highly specific nature of tanycyte messages. Our understanding of the functional effects tanycyte messages is only preliminary and major steps for elucidating their role, remain to be taken.

Very recently, melanocortin-concentrating-hormone-(MCH) immunoreactive ependymal cells, extending between the floor of the 4^th ^ventricle and the midbrain raphe nuclei have been reported [182]. The authors concluded that these elongated tanycytes internalized MCH released from the hypothalamus into the CSF, to act on specific target neurons in the dorsal raphe nucleus. Since serotonergic neurons are numerous in this midbrain raphe nucleus, internalization of MCH followed by release from these specific tanycytes may have far-reaching effects on serotonergic transmission and therefore on behavior.

Additionally, neurons may directly contact the CSF [31,32]. Such neurons have been observed at a considerable distance from the ventricular system. Some use selective receptor-mediated uptake mechanisms to obtain specific substances from the CSF and have been observed in brain areas such as the hippocampus, septum and cortex [170]. Other CSF-contacting neurons were observed in anterodorsal thalamus, supramammillary nucleus, dorsal raphe nucleus, the floor of the fourth ventricle as well as in the lateral superior olive nucleus [178]. This implies, for example, that if the supramammillary region is functionally affected by the CSF contents, the activity of the whole septohippocampal complex is involved, linking functional (learning) and behavioral (fear/anxiety) consequences to the composition of the CSF. In particular, the dorsal raphe nucleus seems to have strong links with the CSF, with several CSF-contacting groups of neurons [178,183] in addition to the special tanycytes, mentioned before. Recently, it was shown that the locus coeruleus accumulates about 50% of a nerve growth factor, not by neuronal transport mechanisms but directly from the CSF [184]. Taken together with the special MCH-transmitting ependymal cells, [182], the activity of the raphe neurons or the serotonergic system, as well as the locus coeruleus neurons or the noradrenergic system, are under control of factors circulating in the CSF. Considering that most of these findings are very recent, it is only to be expected that during the next few years more brain areas and more functional and transmitter systems will be discovered that derive part of their controlling information from CSF.

In summary, the interaction between the CSF and its contents occurs in three different but complementary ways: 1) diffusion: a slow and local process, over short distances, 2) via perivascular spaces and an active overall pump system driven by the vascular system, and 3) specific uptake mechanisms: depending on the receptors exposed to the CSF.

The existence of these mechanisms fully supports the original notion of Borison *et al*, (1980) that "the simple concept that the subarachnoid space constitutes a catch basin for interstitial fluid is no longer tenable. Rather, the CSF of that space must be considered as being in dynamic chemical communication with all parts of the central nervous system" [25]. These mechanisms are in full agreement with the conclusions drawn by Abbott after a careful discussion of available data about interstitial fluid (ISF = ECF) flow, including the CSF [185] "Chemical signals produced within the brain are able to use the flowing ECF (= ISF) as a communication route", with rapid bulk flow occurring within the CSF system, ... "or vectorial signalling from upstream to downstream sites". These mechanisms are ... "extending the sphere of influence of a bioactive molecule beyond that which could be achieved by diffusion"... "and provide the basis for a site specific communication pathway, by which molecules released from site A could be specifically designed to be carried to and influence site B" (pp 550,551). Specificity and range would be determined by factors like the anatomy of the communication route, by the presence of appropriate receptors and by activation mechanisms to limit or confine the signal spread [185]. Abbott (2004) concludes that "the ability to predict the effects of ECF/ISF flow ... is likely to play an important role in developing future therapeutic strategies for CNS targeting and repair".

#### Neuropeptides form an integral part of the CSF composition

In the preceding sections, it has been mentioned that many neuropeptides have been found in the CSF. For many of them, CSF levels bear hardly any relationship to peripheral levels in the blood, and they are actively secreted by choroid plexus, glial and neuronal cells into the CSF [36]. Active release of neuropeptides from brain areas bordering the ventricular system with the aim or at least the effect of influencing downstream brain areas at a distance, has been repeatedly observed for a range of different neuropeptides and transmitters [12,14,36,42,99,102,108,123,129-131,136,184,186-188]. Sewards and Sewards [28] discussed extensively the evidence available for the neuropeptides vasopressin and corticotrophin-releasing-hormone, and concluded that these were actively secreted into the CSF to influence downstream receptive areas involved in specific kinds of behavior.

An interesting recent paper [189] showed the existence of numerous nanoparticles in the CSF, composing a "matrix of membrane- and protein rich nano-scaled structures with many transduction components bounded by lipid membranes". They concluded that this "bulk flow of nanostructures generates a more dispersed signal delivery, of longer duration", in order to "regulate brain behaviors known to require slower, more gradual, and more sustained modulations, such as reported for sleep, appetite, mood and vasomotor regulation". This conclusion is in full agreement with our hypothesis as proposed in the present review.

In summary, there are convincing indications for neuropeptide release into the CSF, and for transport via the CSF bulk flow as a special kind of volume transmission towards receptive brain areas, to propose a message function for specific CSF contents.

### Behavioral effects of CSF contents

The question that remains is: what are the functional significances and the specific contributions of such relatively uncontrolled and broad-spectrum CSF-release systems, in addition to precisely targeted synaptic or more diffuse non-synaptic release mechanisms? Many instances have been discussed above such as melatonin, GnRH, feeding experiments with administration into the 3^rd ^or 4^th ^ventricle, that provide evidence for peptidergic and other signals that arrive at their target areas via the CSF at a concentration level sufficient to induce obvious behavioral changes. Taken together, with the many papers reporting that administration of substances into CSF is much more effective than peripheral administration for inducing behavioral changes, we judge the evidence as overwhelming for the statement that the CSF (the third circulation) contains meaningful messages to be recognized by the appropriate sensitive brain target areas to elicit a neural and physiological/behavioral response. Therefore, we support the view that broadcasting messages via the CSF is an appropriate way to put several parts of the CNS into a behavioral state. Such a brain state is subservient to the performance of specific behaviors or groups of behavioral elements that share a common motivational system and may include the temporary suppression of specific behavioral or physiological reactions. These CSF messages have to be considered as a partially-independent signal. Simultaneously, these messages are constantly supporting, sustaining and in mutual exchange with the ECF contents released by axon terminals all over the brain and specifically in brain areas adjoining the CSF.

Sewards and Sewards [28] proposed that the presence of elevated CSF levels of CRF are involved in the motivation of fear while the presence of vasopressin is involved in the power-dominance drive motivation. They hypothesized that the elevated neuropeptide levels in the CSF are detected and transduced into neuronal activities by, predominantly, hypothalamic neurons in the vicinity of the third ventricle. The purpose of this system was proposed to maintain a state of fear or anger and consequent vigilant or aggressive behavior, after the initial fear- or anger-inducing stimulus itself is no longer perceptible [27]. In other words, CSF messages prolong the duration of a motivated state. Interestingly, important destination areas for this transmission (the medial hypothalamus, periaqueductal gray, midline thalamus and medial) prefrontal cortex are all bordering the CSF system, at the inner or outer surface of the brain. In addition, each of these destination areas is extensively connected to other parts of the limbic system and functionally involved in a range of differently motivated behaviors [27].

The time scale of these effects, starting within minutes but extending over time [57] up to more than an hour [190,191] are fully appropriate to induce and sustain behavioral changes reflecting a modified behavioral state of the animal. The anatomical observations of many varicose peptidergic fibers present close to and in parallel to the ventricular system [12-15,22] suggests that messages arriving via the axons by terminal and varicosity release may be supported by messages arriving via the CSF in a mutually sustaining way, because of the free exchange between ECF and CSF. Such sustaining CSF messages in harmonic balance with neuronal communication mechanisms are in a perfect position to play a role in the rise and decline of successive motivational states and for necessary transitional states.

## Conclusions

There is considerable evidence that peptidergic and other substances are actively secreted into and distributed via the CSF spreading with surprising speed to many brain areas. We propose that they can be considered functionally as messages, because their presence can influence the activity of specific groups of receptive neurons, located near the ventricular and subarachnoid surfaces of the brain, downstream from the site of release. As such messages remain active considerably longer than a few seconds, they may form part of the mechanisms underlying the changes in successive behavioural states.

## Competing interests

The authors declare that they have no competing interests.

## Authors' contributions

JV carried out most of the writing and composition of the paper and the preceding research. HB made substantial contributions to the conception and design of the paper, to the interpretation of the reviewed findings and its critical revision. All authors have read and approved the final version of the manuscript.
